# Tetra­kis(μ-acetato-κ^2^ *O*:*O*′)bis­[(tetra­hydro­furan-κ*O*)chromium(II)]

**DOI:** 10.1107/S2414314623008015

**Published:** 2023-09-22

**Authors:** Christian Heiser, Kurt Merzweiler

**Affiliations:** a Martin-Luther-Universität Halle-Wittenberg, Naturwissenschaftliche Fakultät II, Institut für Chemie, D-06099 Halle, Germany; Vienna University of Technology, Austria

**Keywords:** crystal structure, chromium, acetate, tetra­hydro­furane, paddle wheel

## Abstract

The title compound, [Cr_2_(C_2_H_3_O_2_)_4_(C_4_H_8_O)_2_] or [Cr_2_(OAc)_4_(THF)_2_] (OAc is acetate, THF is tetra­hydro­furan), was obtained by recrystallization of anhydrous chromium(II) acetate [Cr_2_(OAc)_4_] from hot tetra­hydro­furan. The centrosymmetric complex forms monoclinic crystals, space group *C*2/*c*, and consists of two Cr^II^ atoms bridged by four acetate ligands. Additionally, each Cr^II^ atom is coordinated by a terminal THF ligand, which leads to a square-pyramidal coordination.

## Abstract 2

Centrosymmetric [Cr_2_(OAc)_4_(THF)_2_] consists of two Cr^II^ atoms that are bridged by four acetate ions to yield a typical paddle-wheel structure. Furthermore, each chromium atom bears an axially bound THF ligand to give a square-pyramidal coordination.

## Graphical abstract

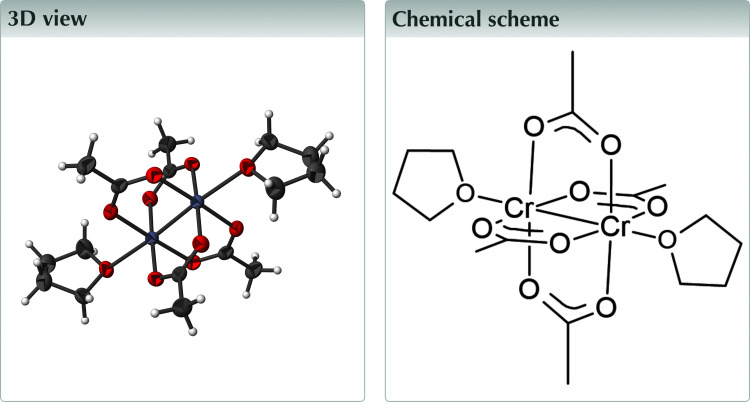

## Structure description

Chromium(II) acetate was discovered as early as 1844 by Peligot (Peligot, 1844 ▸). Determinations of the crystal structure of the dihydrate date back to 1953 (van Niekerk *et al.*, 1953 ▸) and 1971 (Cotton *et al.*, 1971 ▸). A few years later, the crystal structure of anhydrous chromium(II) acetate was reported (Cotton *et al.*, 1977 ▸). Chromium(II) acetate is frequently used as the starting compound for chromium(II) complexes (Cotton *et al.*, 2005 ▸). Over the past decades, a large number of chromium(II) acetate complexes with different ligands *L* have been investigated. Typical compounds are of the type [Cr_2_(OAc)_4_ *L*_2_]. In most cases, *L* represents a nitro­gen ligand such as pyridine (Cotton & Felthouse, 1980 ▸), aceto­nitrile (Cotton *et al.*, 2000 ▸) or 4,4′-bi­pyridine (Cotton & Felthouse, 1980 ▸). However, there are also examples with oxygen donor ligands, among them the dihydrate [Cr_2_(OAc)_4_(H_2_O)_2_] (van Niekerk *et al.*, 1953 ▸) and the analogous derivative with acetic acid ligands [Cr_2_(OAc)_4_(HOAc)_2_] (Cotton & Rice, 1978 ▸). Crystal structures of chromium(II) acetate complexes with common ether donor ligands have not yet been reported. This is in contrast to other chromium(II) carboxyl­ates, where 18 complexes with ether donors have been characterized by crystal-structure determinations. Apart from some di­meth­oxy­ethane (DME) and diethyl ether complexes such as [Cr_2_(9-anthracene­carboxyl­ate)_4_(DME)]_*n*_ (Cotton *et al.*, 1978 ▸) and [Cr_2_(OOC—CF_3_)_4_(OEt_2_)_2_] (Cotton *et al.*, 1978 ▸), this area is dominated by THF complexes. [Cr_2_{OOC—CH(PPh_2_)_2_}_4_(THF)_2_] (Kulangara *et al.*, 2012 ▸), [Cr_2_(OOC—CPh_3_)_4_(THF)_2_] (Cotton & Thompson, 1981 ▸) and [Cr_2_(OOC—C_6_H_4_-*p*-F)_4_(THF)_2_] (Huang *et al.*, 2019 ▸) may serve as representative examples.

Here we report on the crystal structure of [Cr_2_(OAc)_4_(THF)_2_] (**1**). Compound **1** was synthesized by dissolution of anhydrous chromium(II) acetate in hot THF. Upon cooling to room temperature, the product precipitated in the form of dark-red crystals that easily loose THF when separated from the mother liquor.

**Figure 1:**
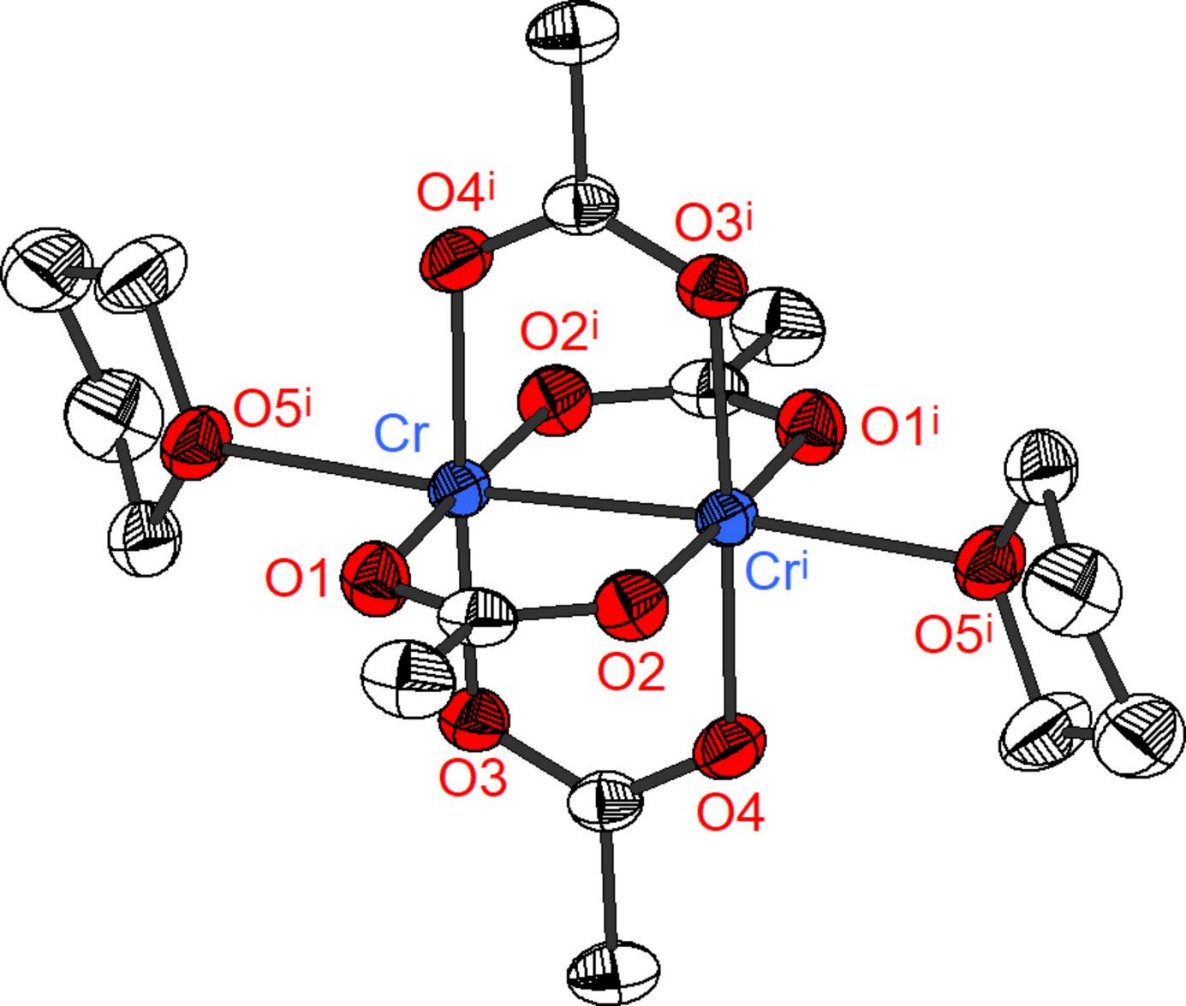
Mol­ecular structure of **1** in the crystal. Displacement ellipsoids are drawn at the 50% probability level. H atoms are omitted for clarity.

The crystal structure of **1** consists of discrete [Cr_2_(OAc)_4_(THF)_2_] mol­ecules that possess crystallographic 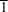 symmetry. The {Cr_2_(OAc)_4_} core displays a characteristic paddle-wheel structure as was observed in the prototypes [Cr_2_(OAc)_4_] (Cotton *et al.*, 1977 ▸) and [Cr_2_(OAc)_4_(H_2_O)_2_] (van Niekerk *et al.*, 1953 ▸). Apart from four acetate O atoms, each Cr^II^ atom binds to the O atom of one THF ligand. This leads to a square-pyramidal coordination environment for the Cr^II^ atoms. A Cr—Cr contact completes the coordination sphere (Fig. 1 ▸). Compound **1** exhibits Cr—O_(OAc)_ distances in the range from 2.0083 (13) to 2.0175 (13) Å (Table 1 ▸). The O_(OAc)_—Cr—O_(OAc)_ angles are 89.37 (6)–90.40 (6)° for the *cis* arranged O atoms and 177.16 (5)–177.24 (5)° for the *trans* positions. The observed bond lengths and angles are typical for [Cr_2_(OAc)_4_ *L*_2_] compounds. According to the Cambridge Structural Database (Groom *et al.*, 2016 ▸), the Cr—O_(OAc)_ distances vary from 1.988 to 2.036 Å with a median value of 2.014 Å (14 entries, 34 data). The *cis-*O_(OAc)_—Cr—O_(OAc)_ angles range between 87.13 and 92.06° with a median of 89.80° (13 entries, 66 data) and the *trans*-O_(OAc)_—Cr—O_(OAc)_ angles are distributed between 173.76 and 178.99° with a median value of 176.65° (14 entries, 25 data).

The Cr—O_(THF)_ distance is 2.3267 (13) Å. [Cr_2_(OAc)_4_(H_2_O)_2_] (Cotton *et al.*, 1971 ▸) and [Cr_2_(OAc)_4_(HOAc)_2_] (Cotton & Rice, 1978 ▸) exhibit corresponding Cr—O distances of 2.272 (3) and 2.306 (3) Å, respectively, for the axially bound ligand. Chromium(II) carboxyl­ates with THF ligands show Cr—O_(THF)_ distances from 2.228 to 2.316 Å with a median of 2.258 Å (14 entries, 14 data).

Compound **1** displays a Cr—Cr distance of 2.3242 (6) Å. This is very close to the median value of 2.337 Å that was obtained from 16 data (14 entries) of the CSD database. Generally, the Cr—Cr distances in [Cr_2_(OAc)_4_ *L*_2_] complexes vary over a relatively large range from 2.270 to 2.452 Å. In [Cr_2_(OAc)_4_(H_2_O)_2_] (Cotton *et al.*, 1971 ▸) and [Cr_2_(OAc)_4_(HOAc)_2_] (Cotton & Rice, 1978 ▸), the Cr—Cr distances are 2.362 (1) and 2.300 (1) Å**.**

**Figure 2:**
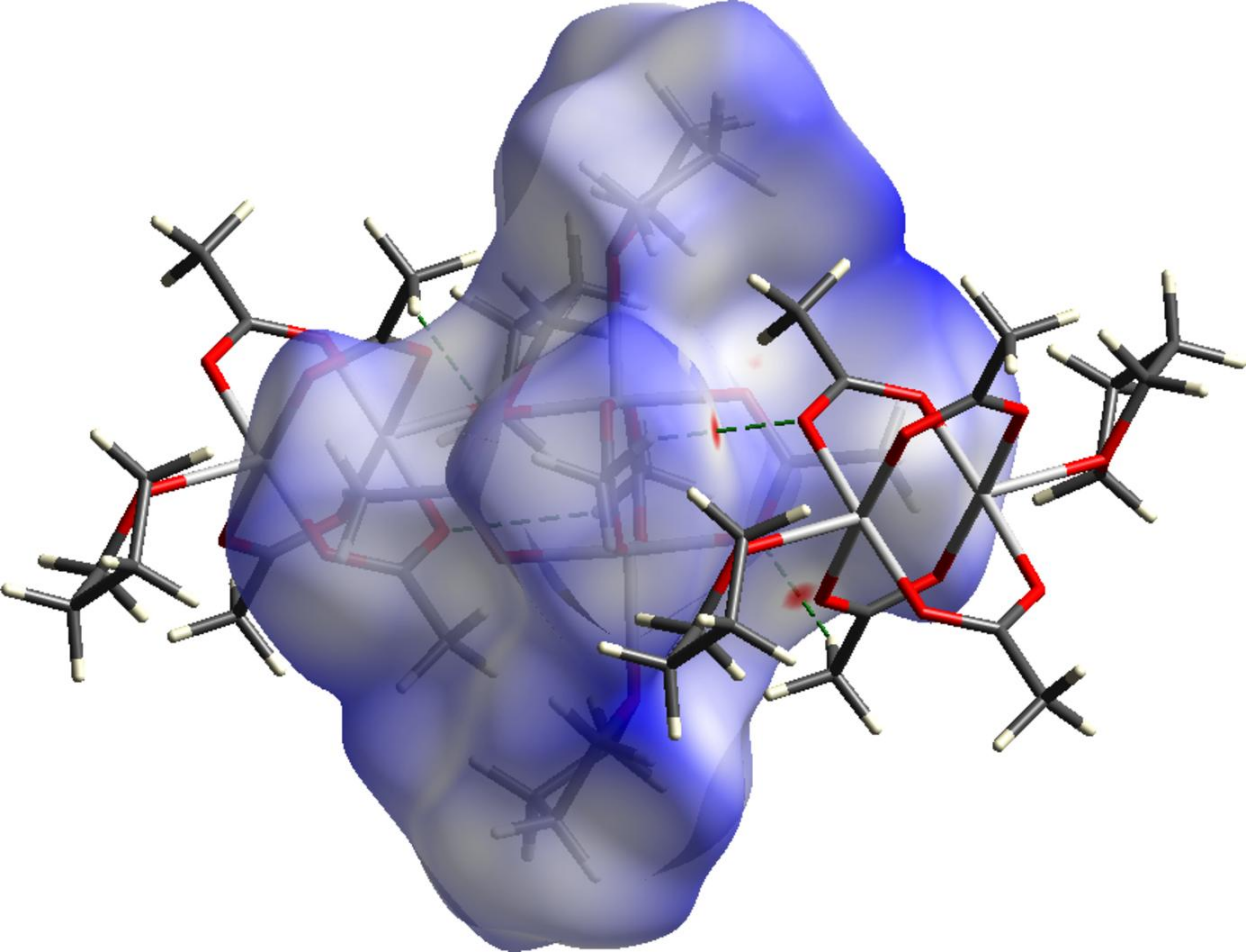
View of the Hirshfeld surface of **1** mapped over *d*_norm_ in the range of −0.062 to 1.826 au. Red-colored surfaces show short contacts, dashed green lines indicate hydrogen-bonding inter­actions.

**Figure 3:**
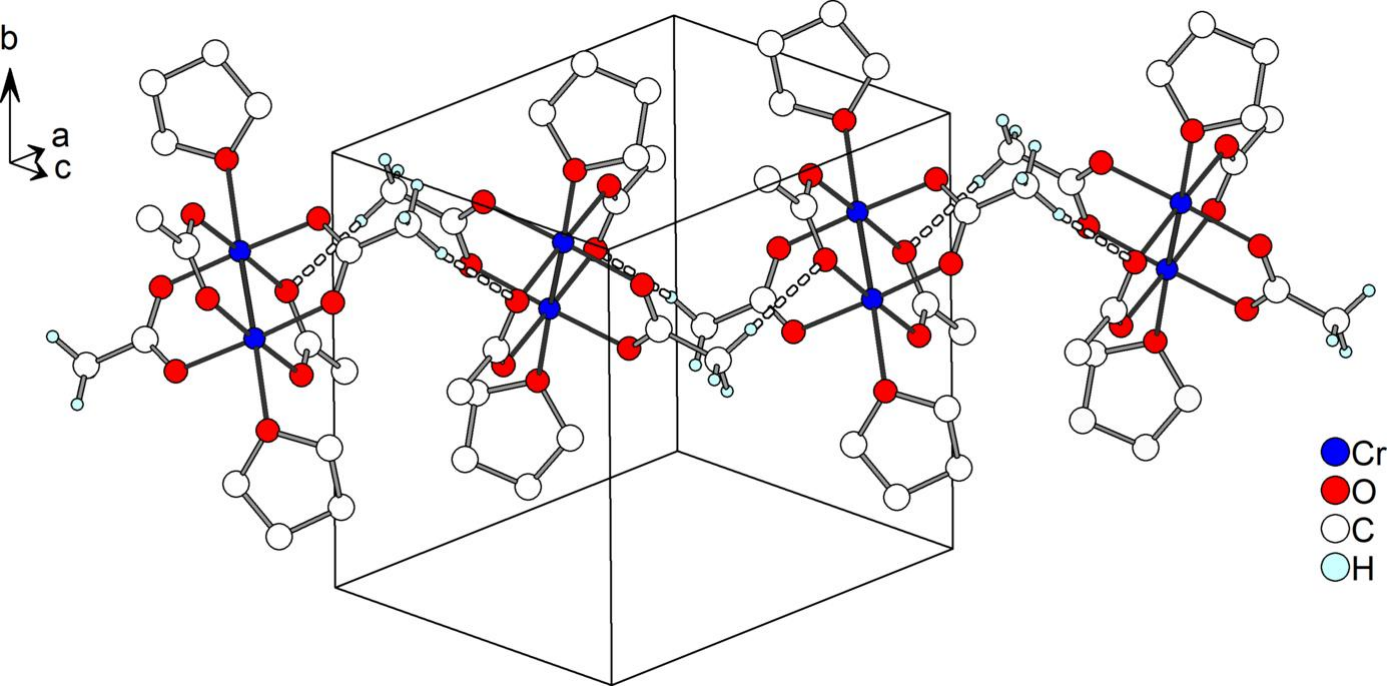
Crystal structure of **1**, with inter­molecular C—H⋯O hydrogen bonds shown as dashed lines.

Regarding supra­molecular inter­actions, a Hirshfeld surface analysis with *CrystalExplorer* (Spackman *et al.*, 2021 ▸) reveals weak C—H⋯O inter­actions (Table 2 ▸) between the acetate methyl group and acetate O atoms of neighbouring mol­ecules (Fig. 2 ▸). As a result, linear chains along [101] are formed (Fig. 3 ▸).

## Synthesis and crystallization

A suspension of chromium(II) acetate (0.5 g; 1.5 mmol) in THF (20 ml) was refluxed for 2 h. Afterwards, the hot solution was filtered and the solid residue further extracted with hot THF (2 × 5 ml). THF was evaporated under reduced pressure to give 20 ml of a concentrated solution. Upon storage at 248 K, the product precipitated after several days. The crystalline compound was filtered off and dried under reduced pressure. Yield: 0.57 g (80%). The chromium content was determined photometrically as chromate (Lange & Vejdělek, 1978 ▸). Analysis for C_16_H_28_Cr_2_O_10_ (484.38): calculated: Cr 21.5%, found: Cr 21.7%; IR (ATR; in cm^−1^): ν = 2962 *w*, 2937 *w*, 2896 *w*, 2867 *w*, 1581 *m*, 1482 *m*, 1435 *s*, 1351 *m*, 1297 *m*, 1249 *w*, 1233 *w*, 1178 *w*, 1035 *m*, 950 *m*, 916 *m*, 878 *m*, 672 *s*, 626 *m*, 583 *m*, 557 *m*, 542 *m*, 495 *m*, 395 *s*, 346 *m*, 297 *s*, 276 *m*, 229 *m*, 208 *m*.

## Refinement

Crystal data, data collection and structure refinement details are summarized in Table 3 ▸.

## Supplementary Material

Crystal structure: contains datablock(s) I. DOI: 10.1107/S2414314623008015/wm4196sup1.cif

Structure factors: contains datablock(s) I. DOI: 10.1107/S2414314623008015/wm4196Isup2.hkl

CCDC reference: 2294928

Additional supporting information:  crystallographic information; 3D view; checkCIF report

## Figures and Tables

**Table 1:** Selected geometric parameters (Å, °)

Cr—Cr^i^	2.3242 (6)	O4—C3	1.261 (2)
Cr—O4^i^	2.0121 (14)	O2—C1	1.262 (2)
Cr—O2^i^	2.0146 (13)	O1—C1	1.263 (2)
Cr—O1	2.0083 (13)	O5—C5	1.447 (2)
Cr—O5	2.3267 (13)	O5—C8	1.444 (2)
Cr—O3	2.0175 (13)	O3—C3	1.262 (2)
			
O4^i^—Cr—O2^i^	90.40 (6)	O1—Cr—O4^i^	89.91 (6)
O4^i^—Cr—O5	89.29 (5)	O1—Cr—O2^i^	177.24 (5)
O4^i^—Cr—O3	177.16 (5)	O1—Cr—O5	94.38 (5)
O2^i^—Cr—O5	88.36 (5)	O1—Cr—O3	90.19 (6)
O2^i^—Cr—O3	89.37 (6)	O3—Cr—O5	93.53 (5)

Symmetry code: (i) 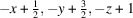 .

**Table 2:** Hydrogen-bond geometry (Å, °)

*D*—H⋯*A*	*D*—H	H⋯*A*	*D*⋯*A*	*D*—H⋯*A*
C4—H4*B*⋯O1^ii^	0.98	2.60	3.472 (3)	148

Symmetry code: (ii) 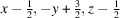 .

**Table 3:** Experimental details

Crystal data	
Chemical formula	[Cr_2_(C_2_H_3_O_2_)_4_(C_4_H_8_O)_2_]
*M*_r_	484.38
Crystal system, space group	Monoclinic, *C*2/*c*
Temperature (K)	213
*a*, *b*, *c* (Å)	20.833 (4), 9.6413 (15), 15.654 (3)
β (°)	136.283 (10)
*V* (Å^3^)	2172.9 (7)
*Z*	4
Radiation type	Mo *K*α
μ (mm^−1^)	1.05
Crystal size (mm)	0.19 × 0.16 × 0.14

Data collection	
Diffractometer	Stoe IPDSII
Absorption correction	Integration [Absorption correction with *X-RED32* (Stoe, 2009 ▸) by Gaussian integration analogous to Coppens (1970 ▸)]
*T*_min_, *T*_max_	0.736, 0.873
No. of measured, independent and observed [*I* > 2σ(*I*)] reflections	8042, 2297, 2085
*R*_int_	0.025
(sin θ/λ)_max_ (Å^−1^)	0.634

Refinement	
*R*[*F* ^2^ > 2σ(*F* ^2^)], *wR*(*F* ^2^), *S*	0.028, 0.084, 1.06
No. of reflections	2297
No. of parameters	129
H-atom treatment	H-atom parameters constrained
Δρ_max_, Δρ_min_ (e Å^−3^)	0.54, −0.25

Computer programs: *X-AREA* (Stoe, 2016 ▸), *SHELXT* (Sheldrick, 2015*a* ▸), *SHELXL* (Sheldrick, 2015*b* ▸), *DIAMOND* (Brandenburg, 2019 ▸ and *OLEX2* (Dolomanov *et al.*, 2009 ▸).

## full crystallographic data

### Crystal data

**Table:**

[Cr_2_(C_2_H_3_O_2_)_4_(C_4_H_8_O)_2_]	*F*(000) = 1008
*M**_r_* = 484.38	*D*_x_ = 1.481 Mg m^−^^3^
Monoclinic, *C*2/*c*	Mo *K*α radiation, λ = 0.71073 Å
*a* = 20.833 (4) Å	Cell parameters from 10024 reflections
*b* = 9.6413 (15) Å	θ = 1.9–27.1°
*c* = 15.654 (3) Å	µ = 1.05 mm^−^^1^
β = 136.283 (10)°	*T* = 213 K
*V* = 2172.9 (7) Å^3^	Block, clear red
*Z* = 4	0.19 × 0.16 × 0.14 mm

### Data collection

**Table:**

Stoe IPDSII diffractometer	2297 independent reflections
Radiation source: sealed X-ray tube, 12 x 0.4 mm long-fine focus	2085 reflections with *I* > 2σ(*I*)
Plane graphite monochromator	*R*_int_ = 0.025
Detector resolution: 6.67 pixels mm^-1^	θ_max_ = 26.8°, θ_min_ = 2.5°
rotation method, ω scans	*h* = −26→26
Absorption correction: integration [Absorption correction with X-Red32 (Stoe, 2009) by Gaussian integration analogous to Coppens (1970)]	*k* = −12→11
*T*_min_ = 0.736, *T*_max_ = 0.873	*l* = −19→19
8042 measured reflections	

### Refinement

**Table:**

Refinement on *F*^2^	0 restraints
Least-squares matrix: full	Hydrogen site location: inferred from neighbouring sites
*R*[*F*^2^ > 2σ(*F*^2^)] = 0.028	H-atom parameters constrained
*wR*(*F*^2^) = 0.084	*w* = 1/[σ^2^(*F*_o_^2^) + (0.0467*P*)^2^ + 2.3382*P*] where *P* = (*F*_o_^2^ + 2*F*_c_^2^)/3
*S* = 1.06	(Δ/σ)_max_ = 0.001
2297 reflections	Δρ_max_ = 0.54 e Å^−^^3^
129 parameters	Δρ_min_ = −0.25 e Å^−^^3^

### Special details

**Table:**

Geometry. All esds (except the esd in the dihedral angle between two l.s. planes) are estimated using the full covariance matrix. The cell esds are taken into account individually in the estimation of esds in distances, angles and torsion angles; correlations between esds in cell parameters are only used when they are defined by crystal symmetry. An approximate (isotropic) treatment of cell esds is used for estimating esds involving l.s. planes.

### Fractional atomic coordinates and isotropic or equivalent isotropic displacement parameters (Å^2^)

**Table:**

	*x*	*y*	*z*	*U*_iso_*/*U*_eq_	
Cr	0.26094 (2)	0.66145 (3)	0.46117 (2)	0.02544 (11)	
O4	0.14551 (9)	0.92201 (13)	0.36838 (12)	0.0342 (3)	
O2	0.34181 (9)	0.94502 (13)	0.57992 (12)	0.0333 (3)	
O1	0.36182 (9)	0.77547 (14)	0.50418 (12)	0.0345 (3)	
O5	0.28145 (10)	0.47338 (14)	0.38969 (12)	0.0372 (3)	
O3	0.16586 (9)	0.75243 (13)	0.29340 (11)	0.0323 (3)	
C1	0.38244 (12)	0.8921 (2)	0.55545 (16)	0.0318 (4)	
C7	0.2675 (2)	0.2300 (2)	0.3690 (3)	0.0598 (7)	
H7A	0.256700	0.153198	0.399458	0.072*	
H7B	0.302134	0.193893	0.352458	0.072*	
C4	0.05644 (14)	0.9286 (2)	0.15474 (18)	0.0437 (5)	
H4A	0.028412	1.008695	0.156494	0.065*	
H4B	0.008049	0.860582	0.094730	0.065*	
H4C	0.086525	0.959150	0.130761	0.065*	
C6	0.17708 (19)	0.2950 (3)	0.2548 (2)	0.0557 (6)	
H6A	0.149691	0.245473	0.179140	0.067*	
H6B	0.131657	0.296526	0.258606	0.067*	
C2	0.46056 (14)	0.9713 (2)	0.5899 (2)	0.0447 (5)	
H2A	0.451210	1.070872	0.590081	0.067*	
H2B	0.461851	0.951885	0.529713	0.067*	
H2C	0.519618	0.942836	0.672441	0.067*	
C5	0.20714 (15)	0.4391 (2)	0.25966 (19)	0.0396 (4)	
H5A	0.229504	0.441548	0.221381	0.048*	
H5B	0.154969	0.505239	0.215539	0.048*	
C3	0.12762 (12)	0.86336 (19)	0.28110 (16)	0.0296 (4)	
C8	0.31894 (19)	0.3462 (2)	0.4601 (2)	0.0537 (6)	
H8A	0.310317	0.343141	0.514527	0.064*	
H8B	0.386159	0.339342	0.512128	0.064*	

### Atomic displacement parameters (Å^2^)

**Table:**

	*U*^11^	*U*^22^	*U*^33^	*U*^12^	*U*^13^	*U*^23^
Cr	0.02569 (16)	0.02424 (17)	0.02535 (16)	0.00141 (10)	0.01810 (14)	0.00139 (10)
O4	0.0342 (7)	0.0303 (6)	0.0301 (6)	0.0073 (5)	0.0206 (6)	0.0046 (5)
O2	0.0330 (6)	0.0300 (6)	0.0358 (7)	−0.0039 (5)	0.0245 (6)	−0.0014 (5)
O1	0.0324 (6)	0.0367 (7)	0.0387 (7)	−0.0011 (5)	0.0272 (6)	−0.0005 (6)
O5	0.0429 (7)	0.0283 (6)	0.0389 (7)	0.0034 (6)	0.0290 (6)	−0.0013 (5)
O3	0.0353 (6)	0.0321 (6)	0.0276 (6)	0.0030 (5)	0.0221 (6)	0.0024 (5)
C1	0.0269 (8)	0.0356 (9)	0.0257 (8)	−0.0001 (7)	0.0167 (7)	0.0068 (7)
C7	0.090 (2)	0.0343 (11)	0.0689 (16)	0.0016 (12)	0.0617 (16)	0.0012 (11)
C4	0.0388 (10)	0.0433 (11)	0.0299 (9)	0.0054 (9)	0.0185 (9)	0.0106 (8)
C6	0.0637 (15)	0.0443 (12)	0.0557 (14)	−0.0147 (11)	0.0420 (13)	−0.0109 (11)
C2	0.0333 (10)	0.0517 (12)	0.0432 (11)	−0.0084 (9)	0.0257 (9)	0.0034 (9)
C5	0.0444 (11)	0.0372 (10)	0.0373 (10)	0.0029 (8)	0.0296 (9)	−0.0014 (8)
C3	0.0245 (8)	0.0303 (8)	0.0264 (8)	−0.0014 (7)	0.0159 (7)	0.0038 (7)
C8	0.0604 (14)	0.0347 (11)	0.0486 (13)	0.0132 (10)	0.0336 (12)	0.0067 (9)

### Geometric parameters (Å, º)

**Table:**

Cr—Cr^i^	2.3242 (6)	C7—C8	1.492 (3)
Cr—O4^i^	2.0121 (14)	C4—H4A	0.9800
Cr—O2^i^	2.0146 (13)	C4—H4B	0.9800
Cr—O1	2.0083 (13)	C4—H4C	0.9800
Cr—O5	2.3267 (13)	C4—C3	1.506 (2)
Cr—O3	2.0175 (13)	C6—H6A	0.9900
O4—C3	1.261 (2)	C6—H6B	0.9900
O2—C1	1.262 (2)	C6—C5	1.503 (3)
O1—C1	1.263 (2)	C2—H2A	0.9800
O5—C5	1.447 (2)	C2—H2B	0.9800
O5—C8	1.444 (2)	C2—H2C	0.9800
O3—C3	1.262 (2)	C5—H5A	0.9900
C1—C2	1.501 (3)	C5—H5B	0.9900
C7—H7A	0.9900	C8—H8A	0.9900
C7—H7B	0.9900	C8—H8B	0.9900
C7—C6	1.506 (4)		
			
Cr^i^—Cr—O5	176.08 (4)	H4A—C4—H4C	109.5
O4^i^—Cr—Cr^i^	88.19 (4)	H4B—C4—H4C	109.5
O4^i^—Cr—O2^i^	90.40 (6)	C3—C4—H4A	109.5
O4^i^—Cr—O5	89.29 (5)	C3—C4—H4B	109.5
O4^i^—Cr—O3	177.16 (5)	C3—C4—H4C	109.5
O2^i^—Cr—Cr^i^	88.66 (4)	C7—C6—H6A	111.4
O2^i^—Cr—O5	88.36 (5)	C7—C6—H6B	111.4
O2^i^—Cr—O3	89.37 (6)	H6A—C6—H6B	109.2
O1—Cr—Cr^i^	88.61 (4)	C5—C6—C7	101.9 (2)
O1—Cr—O4^i^	89.91 (6)	C5—C6—H6A	111.4
O1—Cr—O2^i^	177.24 (5)	C5—C6—H6B	111.4
O1—Cr—O5	94.38 (5)	C1—C2—H2A	109.5
O1—Cr—O3	90.19 (6)	C1—C2—H2B	109.5
O3—Cr—Cr^i^	88.98 (4)	C1—C2—H2C	109.5
O3—Cr—O5	93.53 (5)	H2A—C2—H2B	109.5
C3—O4—Cr^i^	120.14 (11)	H2A—C2—H2C	109.5
C1—O2—Cr^i^	119.21 (12)	H2B—C2—H2C	109.5
C1—O1—Cr	119.57 (12)	O5—C5—C6	105.37 (17)
C5—O5—Cr	118.23 (11)	O5—C5—H5A	110.7
C8—O5—Cr	118.74 (13)	O5—C5—H5B	110.7
C8—O5—C5	108.56 (15)	C6—C5—H5A	110.7
C3—O3—Cr	118.98 (11)	C6—C5—H5B	110.7
O2—C1—O1	123.94 (17)	H5A—C5—H5B	108.8
O2—C1—C2	118.42 (18)	O4—C3—O3	123.71 (16)
O1—C1—C2	117.64 (18)	O4—C3—C4	118.37 (17)
H7A—C7—H7B	109.0	O3—C3—C4	117.92 (17)
C6—C7—H7A	111.0	O5—C8—C7	106.84 (19)
C6—C7—H7B	111.0	O5—C8—H8A	110.4
C8—C7—H7A	111.0	O5—C8—H8B	110.4
C8—C7—H7B	111.0	C7—C8—H8A	110.4
C8—C7—C6	103.9 (2)	C7—C8—H8B	110.4
H4A—C4—H4B	109.5	H8A—C8—H8B	108.6
			
Cr^i^—O4—C3—O3	−0.4 (2)	Cr—O3—C3—O4	0.2 (2)
Cr^i^—O4—C3—C4	179.40 (13)	Cr—O3—C3—C4	−179.60 (13)
Cr^i^—O2—C1—O1	1.2 (2)	C7—C6—C5—O5	−34.4 (2)
Cr^i^—O2—C1—C2	−178.35 (12)	C6—C7—C8—O5	−23.6 (3)
Cr—O1—C1—O2	−1.6 (2)	C5—O5—C8—C7	1.9 (3)
Cr—O1—C1—C2	177.88 (12)	C8—O5—C5—C6	20.7 (2)
Cr—O5—C5—C6	−118.42 (16)	C8—C7—C6—C5	35.1 (3)
Cr—O5—C8—C7	140.80 (18)		

Symmetry code: (i) −*x*+1/2, −*y*+3/2, −*z*+1.

### Hydrogen-bond geometry (Å, º)

**Table:**

*D*—H···*A*	*D*—H	H···*A*	*D*···*A*	*D*—H···*A*
C4—H4*B*···O1^ii^	0.98	2.60	3.472 (3)	148

Symmetry code: (ii) *x*−1/2, −*y*+3/2, *z*−1/2.
